# PrhN, a putative marR family transcriptional regulator, is involved in positive regulation of type III secretion system and full virulence of *Ralstonia solanacearum*

**DOI:** 10.3389/fmicb.2015.00357

**Published:** 2015-04-28

**Authors:** Yong Zhang, Feng Luo, Dousheng Wu, Yasufumi Hikichi, Akinori Kiba, Yasuo Igarashi, Wei Ding, Kouhei Ohnishi

**Affiliations:** ^1^Research Center of Bioenergy and Bioremediation, Southwest UniversityChongqing, China; ^2^College of Plant Protection, Southwest UniversityChongqing, China; ^3^Laboratory of Plant Pathology and Biotechnology, Kochi UniversityKochi, Japan; ^4^Research Institute of Molecular Genetics, Kochi UniversityKochi, Japan

**Keywords:** *R. solanacearum*, type III secretion system, Hrp regulon, pathogenesis, PrhN, MarR type regulator

## Abstract

The MarR-family of transcriptional regulators are involved in various cellular processes, including resistance to multiple antibiotics and other toxic chemicals, adaptation to different environments and pathogenesis in many plant and animal pathogens. Here, we reported a new MarR regulator PrhN, which was involved in the pathogenesis of *Ralstonia solanacearum*. prhN mutant exhibited significantly reduced virulence and stem colonization compared to that of wild type in tomato plants. prhN mutant caused identical hypersensitive response (HR) on resistant plants to the wild type. Deletion of prhN gene substantially reduced the expression of type III secretion system (T3SS) *in vitro* and in planta (mainly in tomato plants), which is essential for pathogenicity of *R. solanacearum*, and the complemented PrhN could restore its virulence and T3SS expression to that of wild type. T3SS is directly controlled by AraC-type transcriptional regulator HrpB, and the transcription of hrpB is activated by HrpG and PrhG. HrpG and PrhG are homologs but are regulated by the PhcA positively and negatively, respectively. Deletion of prhN gene also abolished the expression of hrpB and prhG, but didn't change the expression of hrpG and phcA. Together, these results indicated that PrhN positively regulates T3SS expression through PrhG and HrpB. PrhN and PhcA should regulate prhG expression in a parallel way. This is the first report on the pathogenesis of MarR regulator in *R. solanacearum*, and this new finding will improve our understanding on the various biological functions of MarR regulator and the complex regulatory network on hrp regulon in *R. solanacearum*.

## Introduction

The MarR (multiple antibiotic resistance regulator) protein was originally identified as repressor of the multiple antibiotic resistance operon *marRAB* in *Escherichia coli*. It is a prototypical member of the MarR-family of transcriptional regulators (Seoane and Levy, 1995; Wilkinson and Grove, 2006). MarR members are widely distributed in many bacteria and archaea and are involved in various cellular processes, including resistance to multiple antibiotics and other toxic chemicals, adaptation to different environments and the pathogenesis of many plant and animal pathogens (Alekshun et al., 2001; Ellison and Miller, 2006). A majority of MarR members are characterized as transcriptional repressors, several of which are activators, i.e., SlyA is suggested to repress and activate expression of target genes (Stapleton et al., 2002; Haque et al., 2009; Zou et al., 2012). The role of MarR members in pathogenesis is to control the expression of some virulence-related genes or virulence-associated traits (Ellison et al., 2004; Wilkinson and Grove, 2004). It has been recently reported that the MarR regulator SlyA regulates Type III secretion system (T3SS) genes in parallel with the T3SS master regulator HrpL and is important for the virulence of *Dickeya dadantii* 3937 (Haque et al., 2009; Zou et al., 2012). In *Xanthomonas campestris* pathovar campestris (taxonomically close to *Ralstonia solanacearum*), the MarR regulator HpaR is positively controlled by HrpG and HrpX and is involved in pathogenesis (Wei et al., 2007). MarR regulators are conserved in many pathogens, but whether some regulators share MarR-like activities in *R. solanacearum* remains to be characterized.

*R. solanacearum* is one of the most destructive bacterial plant pathogens and currently, there are no generally effective controls for the lethal bacterial wilt diseases caused by *R. solanacearum* (Mansfield et al., 2012; Peeters et al., 2013). *R. solanacearum* is a Gram-negative, soil-borne vascular bacterium, and like in many plant and animal bacterial pathogens, the syringe-like T3SS plays a crucial role in the pathogenicity of *R. solanacearum* (Angot et al., 2006; Galán and Wolf-Watz, 2006). Bacteria use T3SS to inject virulence effectors, so-called type III effectors, into host cells to interfere with cellular defenses (Gohre and Robatzek, 2008; Poueymiro and Genin, 2009). In *R. solanacearum*, the T3SS is encoded by a gene cluster of more than 20 hypersensitive response and pathogenicity (*hrp*) genes; these genes form a cluster known as the *hrp* regulon (Van Gijsegem et al., 1995; Cornelis and Van Gijsegem, 2000). Transcription of *hrp* regulon is repressed in nutrient-rich medium and induced in nutrient-poor medium, which may mimic conditions in the intercellular spaces of plants (Arlat et al., 1992; Coll and Valls, 2013). Plant signals or some mimic signals can induce *hrp* expression at levels to 10 to 20-fold greater than that in nutrient-poor conditions (Jacobs et al., 2012; Monteiro et al., 2012). The T3SS and type III effectors are directly controlled by the AraC-type transcriptional regulator HrpB, which binds directly to the plant-inducible promoter (PIP) motif in the promoter regions of its target genes (Cunnac et al., 2004; Mukaihara et al., 2010). The transcription of *hrpB* is activated by both HrpG and PrhG, which are close paralogs (72% global identity) and belong to the OmpR/PhoB family of two-component response regulators (Plener et al., 2010; Zhang et al., 2013). The regulation of HrpG on *hrpB* expression is activated by some plant-related signals which are perceived by the outer membrane receptor PrhA or some unknown receptors and transduced to HrpG through the PrhA-PrhR/PrhI-PrhJ cascade or some unknown pathway (Valls et al., 2006; Yoshimochi et al., 2009b). PrhG is dispensable for this signaling cascade (Zhang et al., 2013; Zuluaga et al., 2013). HrpG has been well illustrated as master regulator since *hrpG* mutants are impaired in growth *in planta* and complete lost the pathogenicity in host plants (Vasse et al., 2000; Valls et al., 2006; Yoshimochi et al., 2009b). *PrhG* mutant can grow the same as do wild-type bacteria *in planta* and show slightly reduced virulence compared to that of wild-type (Zhang et al., 2013). The global virulence regulator PhcA, which is quorum sensing-dependent, negatively regulates the expression of *hrpG* through PrhIR in an indirect manner (Genin et al., 2005; Yoshimochi et al., 2009a). PhcA positively regulates *prhG* expression (Zhang et al., 2013). In this process, PhcA regulates *hrpB* expression in opposite ways. *R. solanacearum* may switch from using HrpG to PrhG for *hrpB* activation in a cell density-dependent manner (Zhang et al., 2013).

In a previous study using transposon mutagenesis, we isolated some *prh* (positive regulation of *hrp* regulon) genes from the Japanese *R. solanacearum* strain OE1-1 (Zhang et al., 2013). OE1-1 causes disease in tomato and tobacco plants (Kanda et al., 2003). We constructed a *popA-lacZYA* fusion in OE1-1 to monitor the expression profile of the *hrp* regulon, in which the promoterless *lacZYA* operon was integrated downstream of the *popA* gene and shared a promoter with *popA* (a schematic is available as Figure S1 in Zhang et al., 2013). The *popA* gene exists as part of an operon with *popB* and *popC*, which maps to the left-side of the *hrp* regulon. The *popA* operon is directly controlled by HrpB and *popA-lacZYA* exhibits an expression profile that is identical to that of the *hrp* regulon under different conditions. The generated reporter strain RK5050 (OE1-1 *popA-lacZYA*) exhibits bacterial growth and virulence that are identical to OE1-1 in tomato and tobacco plants (Yoshimochi et al., 2009b; Zhang et al., 2011). To better elucidate the regulation mechanism of the *hrp* regulon in *R. solanacearum*, we focused on Rsc1721 (prh18 in Zhang et al., 2013 hereafter designated as PrhN). PrhN contains 188 amino acids and is a putative MarR-type transcriptional regulator (https://iant.toulouse.inra.fr/bacteria/annotation/cgi/ralso.cgi). We analyzed the contribution of PrhN to *hrp* expression and pathogenicity in *R. solanacearum*, and demonstrated genetically that the newly characterized MarR regulator PrhN positively regulates the *hrp* regulon indirectly and is required for full virulence of *R. solanacearum* in host plants.

## Materials and methods

### Bacterial strains and culture conditions

Bacterial strains used in this study are listed in Table 1. *R. solanacearum* strains are derivatives of the strain OE1-1 (phylotype I, race 1, biovar 3) (Kanda et al., 2003) and RS1002 (phylotype I, race 1, biovar 4) (Mukaihara et al., 2004). The OE1-1 strain is pathogenic on tomato and tobacco plants, and the strain RS1002 is pathogenic on tomato plants and elicits a hypersensitive response (HR) in tobacco leaves. *E. coli* strains DH12S and S17-1 (Simon et al., 1983) were used for plasmid construction and conjugational transfer, respectively. *E. coli* was grown in Luria-Bertani (LB) medium at 37°C. *R. solanacearum* was grown at 28°C in rich B medium or in hydroponic plant culture medium containing 2% sucrose (sucrose medium, in which the *hrp* expression can be well induced) (Yoshimochi et al., 2009b; Zhang et al., 2011). Antibiotics were added into the medium at the following concentrations: ampicillin (Ap), 100 μg ml^−1^; gentamycin (Gm), 20 μg ml^−1^; kanamycin (Km), 50 μg ml^−1^; polymyxin B (PB), 50 μg ml^−1^.

**Table 1:** ***R. solanacearum* stains used in this study**.

**Strain**	**Relative characteristics**	**Reference source**
OE1-1	Wild-type, race 1, biovar 3	Kanda et al., 2003
RK5046	OE1-1 *hrpB-lacZYA*	Zhang et al., 2013
RK5050	OE1-1 *popA-lacZYA*	Zhang et al., 2013
RK5120	OE1-1 *hrpG-lacZYA*	Zhang et al., 2013
RK5185	*popA-lacZYA*, Δ*prhG*	Zhang et al., 2013
RK5212	OE1-1 *prhG-lacZYA*	Zhang et al., 2013
RK5234	*prhG-lacZYA*, Δ*phcA*	Zhang et al., 2013
RK5601	*popA-lacZYA*, Δ*prhN*	This work
RK5604	*hrpB-lacZYA*, Δ*prhN*	This work
RK5607	*prhG-lacZYA*, Δ*prhN*	This work
RK5610	*hrpG-lacZYA*, Δ*prhN*	This work
RK5613	OE1-1 *prhN-lacZYA*	This work
RK5619	*prhN-lacZYA, Δ phcA*	This work
RK5701	RK5601 (Δ*prhN*+*prhN*)^c^	This work
RK5704	RK5601 (Δ*prhN*+*prhN*)^c^	This work
RK5707	RK5607 (Δ*prhN*+*prhN*)^c^	This work
RS1002	Wild-type, race 1, biovar 4	Mukaihara et al., 2004
RK10001	RS1002, *popA-lacZYA*	Mukaihara et al., 2004
RK10010	RK10001, Δ*prhN*	This work
RK10013	RK10010 (Δ*prhN*+*prhN*)^c^	This work

c *Complementation with prhN gene (under the control of its native promoter)*.

### Construction of *prhN* deletion mutants

Plasmids designed to create deletion mutants were based on pK18mobsacB (Scḧafer et al., 1994). To construct a complete in-frame deletion of *prhN*, two DNA fragments flanking the *prhN* gene were amplified from OE1-1 genomic DNA using PrimeSTAR HS DNA polymerase (Takara Japan). The left flanking fragment was amplified using the primers rsc1721A1BamHI (5′-CAT**GGATCC**GATGTCGAGTGCGTAGGC-3′) and rsc1721B1C (5′-CCGGCCCGAACGGGCGTTGCGTGACTCGCGGGCAGA-3′). The right flanking fragment was amplified using the primers rsc1721A2C (5′-TCTGCCCGCGAGTCACGCAACGCCCGTTCGGGCCGG-3′) and rsc1721B2HindIII (5′-CAT**AAGCTT**GCGGTCGTGCAGGTGCGC-3′). rsc1721B1C and rsc1721A2C are full complements that contain 18 bases of nucleic aligning to the left and right flanking fragments, respectively. Two DNA fragments (~600-bp) were purified from an agarose gel and subjected to a second round of PCR amplification using the primers rsc1721A1BamHI and rsc1721B2HindIII. Next, a 1.2-kb fragment was agarose gel-purified and cloned using the TA-blunt ligation kit (Nippon gene) into pBluescript II KS (+) vector predigested with *Eco*R-V. The resulting plasmid was named pKSdprhN and the sequence was validated. Next, a *Bam*H-I-*Hin*d-III digested DNA fragment from pKSdprhN was sub-cloned into a *Bam*H-I-*Hin*d-III digested pK18mobsacB vector and pK18dprhN was generated.

After the sequence was validated, the plasmid pK18dprhN was transferred from *E. coli* strain S17-1 into the OE1-1 derivative strains RK5050 (*popA-lacZYA*), RK5046 (*hrpB-lacZYA*), RK5120 (*hrpG*-*lacZYA*), and RK5212 (*prhG-lacZYA*), and the RS1002 derivative strain RK10001 (RS1002, *popA-lacZYA*). The deletion of *prhN* at its original location was confirmed by colony PCR using primers rsc1721A1BamHI and rsc1721B2HindIII (the expected PCR band size for wild type strains was 1.7-kb; PCR band size for *prhN* deletion was 1.2-kb). The *prhN* deletion strains RK5601 (*popA-lacZYA, Δ prhN*), RK5604 (*hrpB-lacZYA, Δ prhN*), RK5607 (*prhG-lacZYA, Δ prhN*), RK5610 (*hrpG*-*lacZYA, Δ prhN*), and RK10010 (RK0001, Δ*prhN*) were obtained.

### Complementation analyses

In the present study, complementation analyses were performed using a Tn*7*-based site-specific integration system. The gene for complementation was cloned into pUC18-mini-Tn7T-Gm vector (Choi et al., 2005; Zhang et al., 2011). The *prhN* gene (containing a 613-bp region upstream of *prhN*, which possibly includes its native promoter) was amplified using primers rsc1721A1BamHI and rsc1721B3HindIII (5′-CAT**AAGCTT**TTACAGCGAGGTGGCCGAAC-3′). The purified ~1.2-kb DNA fragment was cloned using the TA-blunt ligation kit (Nippon gene) into pBluescript II KS (+) predigested with *Eco*R-V and pKSprhNC was constructed. After the sequence was validated, a *Bam*H-I-*Hin*d-III digested DNA fragment from pKSprhNC was sub-cloned into *Bam*H-I-*Hin*d-III digested pUC18-mini-Tn7T-Gm and pUCprhN was generated. After the sequence was validated, the transposase-containing helping plasmid pTNS2 was used to electroporate pUCprhN into *prhN* mutants. The *prhN* gene (containing its possible native promoter) was specifically integrated into a single *att*Tn*7* site (25-bp downstream of the *glmS* gene). Insertion at the *att*Tn*7* site was confirmed by colony PCR using primers glmS-down and Tn7R (Zhang et al., 2011).

### Construction of *prhN-lacZYA* reporter strain and deletion with *phcA*

The promoterless *lacZYA* fragment from pUClacZYA (Yoshimochi et al., 2009b) was inserted 24-bp downstream of the start codon of *prhN*, where six nucleotides (GAAAGT) were replaced with *Bam*H I sequence (GGATCC). Two DNA fragments flanking insertion site were amplified using PCR. The primers rsc1721A4EcoRI (5′-CAT**GAATTC**GATGTCGAGTGCGTAGGC-3′) and rsc1721B4BamHI (5′-TCGTCGTCCTCTTC**GGATCC**CGATTTG-3′) were used to amplify left flanking fragment. rsc1721A4BamHI (5′-CAAATCG**GGATCC**GAAGAGGACGACGA-3′) and rsc1721B2HindIII were used to amplify the right flanking fragment. The primers rsc1721B4BamHI and rsc1721A4BamHI are full complements. Two DNA fragments were purified from agarose gel and subjected for a second round of PCR amplification using primers rsc1721A4EcoRI and rsc1721B4HindIII. The agarose gel-purified 1.2-kb fragment was cloned using the TA-blunt ligation kit (Nippon gene) into pBluescript II KS (+) predigested with *Eco*R-V and pKSprhNB was constructed. After the sequence was validated, a *Eco*R-I-*Hin*d-III digested DNA fragment from pKSprhNB was sub-cloned into *Eco*R-I-*Hin*d-III digested pK18mobsacB vector and pK18prhNB was generated. Subsequently, a *Bam*H-I digested promoterless *lacZYA* fragment from pUClacZYA was inserted into *Bam*H-I digested pK18prhNB with the same direction as *prhN* and pK18prhN-lacZYA was generated.

After the sequence was validated, plasmid pK18prhN-lacZYA was transferred from *E. coli* S17-1 into *R. solanacearum* OE1-1 and RK5613 (OE1-1, *prhN-lacZYA*) was obtained. When the *phcA* deletion was introduced into RK5613, the plasmid phcA22 (used for the *phcA* deletion based on pK18mobsacB, Yoshimochi et al., 2009b) was transferred from *E. coli* strain S17-1 into RK5613, and RK5619 (*prhN-lacZYA, Δ phcA*) was obtained.

### β-galactosidase assay

The *β*-galactosidase assay was performed as described previously (Zhang et al., 2011). Bacterial cells (40 μ l) were used to inoculate 2 ml of fresh medium and incubated with shaking at 28°C for 5 h in B medium or 8 h in sucrose medium (cells where grown to an approximate OD600 of 0.1). When cells were collected at a low density, i.e., at an approximate OD600 of 0.01, 30 μ l of bacterial cells was used to inoculate 40 ml of fresh medium and cells where grown to an approximate OD600 of 0.01 after 5 or 8 h cultivation. Next, 40 ml of cell culture was centrifuged and resuspended into 2 ml of fresh medium. When cells were collected at a high density, i.e., at an approximate OD600 of 1.0, 200 μ l of bacterial cells was used to inoculate 2 ml of fresh medium and cells will grow to an approximate OD600 of 1.0 after 5 or 8 h cultivation. An aliquot (100 or 200 μ l) of cells was used for *β*-galactosidase assay. The enzyme assay was repeated in at least three independent times and mean values were averaged with standard deviation (SD). Statistical significance was determined by the two-tailed Student's *t*-test.

### Virulence assays and HR tests

Virulence assays were performed on wilt-susceptible plants (tomato, *Solanum lycopersicum* cv. Moneymaker and tobacco, *Nicotiana tabacum* cv. Bright Yellow) using the soil-soak and petiole inoculation procedures as described previously (Yao and Allen, 2007; Zhang et al., 2013). For the soil-soak inoculation, 2–3-week-old tomato plants or 3–4-week-old tobacco plants were inoculated with bacteria at a final concentration of 10^7^ cfu g^−1^ of soil and incubated at 25°C under 10000 lx. For petiole inoculation in tomato plants, 2 μ l of bacteria at 10^7^ cfu ml^−1^ was dropped onto the freshly cut surface of petioles. Plants were inspected for wilt symptoms daily over approximately 20 days post inoculation (dpi). Each test for one strain included at least 12 plants per treatment and each assay was repeated in at least four independent trials. The mean values were averaged with SD. For the HR test in tobacco leaves, *N. tabacum* BY leaves were infiltrated with approximately 50 μ l of bacterial suspension at 10^8^ cfu ml^−1^ with a blunt-end syringe. Plants were incubated at 25°C, and HR symptom development was recorded periodically. Each test was repeated at least three times and a representative result is presented.

### Bacterial growth *in planta*

Bacteria *in planta* were collected from stem tissues and quantified by dilution plating as previously described (Zhang et al., 2013). Two-to-three-week-old tomato plants were inoculated with bacteria using the soil-soak procedure and stem pieces (5 cm above the soil and 1 cm in length) were removed at 4 dpi (wilt symptoms reached 1–2) and 7 dpi (wilt symptoms reached 3–4). Each test included at least three samples from different plants and mean values of four independent trials were averaged with SD. Statistical significance was determined by the two-tailed Student's *t*-test.

### β-galactosidase assay in *planta*

The *β*-galactosidase assay *in planta* was performed as described previously (Zhang et al., 2013). Plants (tomato and tobacco) were inoculated with bacteria using petiole inoculation and leaf infiltration procedures, respectively. For petiole inoculation, 2 μ l of bacterial cells (10^8^ cfu ml^−1^) was placed on the freshly cut surface of tomato petioles. For leaf infiltration, approximately 100 μ l of bacterial cells (10^4^ cfu ml^−1^) was infiltrated into tobacco and tomato leaves. After incubation for various times, leaf discs (0.38 cm^2^) and tomato stem pieces (1 cm in length) were removed from plants and bacteria were collected for *β*-galactosidase assay. Enzymatic activity *in planta* was determined using a Galacto-Light Plus kit (Applied Biosystems) and the luminescence was evaluated using GloMax 20/20 luminometer (Promega). Enzymatic activity was normalized with luminescence divided by cell number. Each assay included at least three samples from different plants and mean values of at least four independent trials were averaged with SD. Statistical significance was determined by the two-tailed Student's *t*-test.

### Nucleotide sequence accession number

The nucleotide sequences of *prhN* in strain OE1-1 have been deposited into the DDBJ database under accession number AB981315.

## Results

### PrhN is important for *popA* and *hrpB* expression *in vitro*

Generated by transposon mutagenesis, a *prhN* mutant exhibited substantially reduced *popA* expression, indicating that PrhN is a positive regulator of the *popA* operon in *R. solanacearum* OE1-1 (Zhang et al., 2013). PrhN is well conserved in many bacterial strains in addition to *R. solanacearum*. PrhN in *R. solanacearum* OE1-1 is more than 96% identical to those in *R. solanacearum* strains, approximately 80% identical to those in *Ralstonia* sp. and *Cupriavidus* sp., approximately 60% identical to those in *Chromobacterium* sp. and others. Searching the genome databank of *R. solanacearum* strains, only seldom provides gene products that are presumed to be MarR-type regulators. A genome BLAST search revealed that Rsc1721 is a unique putative MarR-type regulator in GMI1000, whose genome sequence has been completed and well studied (Salanoubat et al., 2002).

**Figure 1:**
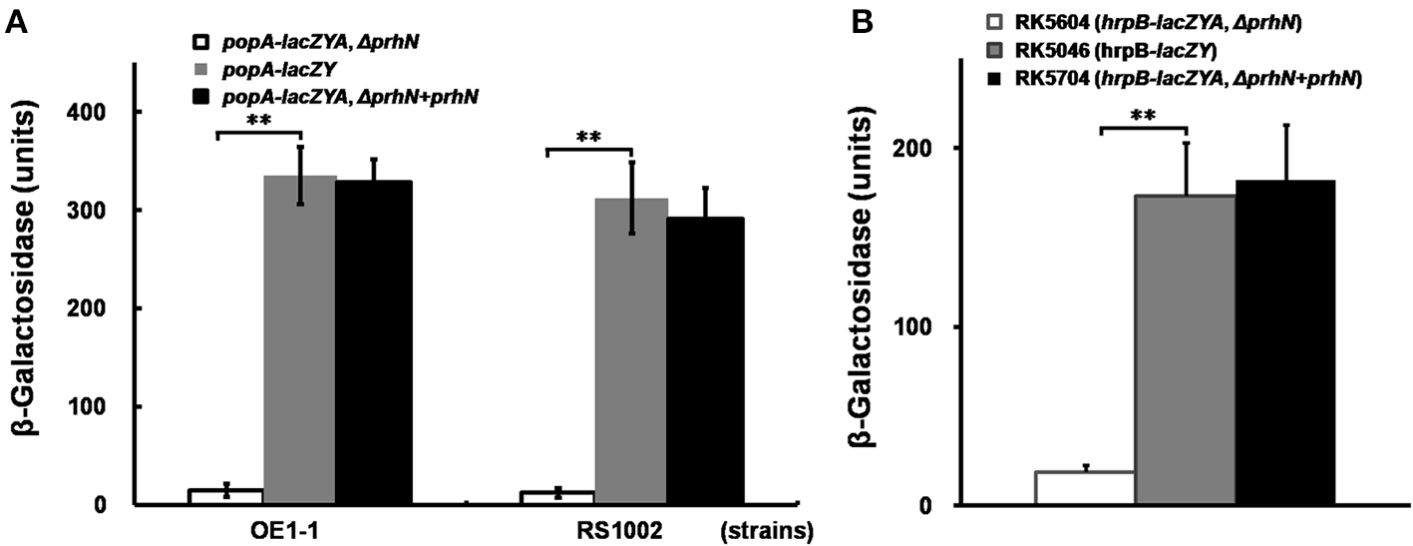
***popA* and *hrpB* expression in *prhN* mutants**. **(A)** *popA*, **(B)** *hrpB* expression in sucrose medium. **(A)** *prhN* mutants (*popA-lacZAY*, Δ*prhN*), white bars; wild type (*popA-lacZAY*), gray bars; and strains with complementation (*popA-lacZAY*, Δ*prhN*+*prhN*), dark bars. Derivatives of strain OE1-1: RK5601 (*prhN* mutant), RK5050 (wild type), and RK5701 (complementation). Derivatives of strain RS1002: RK10010 (*prhN* mutant), RK10001 (wild type), and RK10013 (complementation). **(B)** RK5604 (*hrpB-lacZAY*, Δ*prhN*), white bars; RK5046 (*hrpB-lacZAY*), gray bars and RK5704 (complementation, *hrpB-lacZAY*, Δ*prhN*+*prhN*), dark bars. Cells were grown in sucrose medium to an OD600 of about 0.1, which corresponds to 1.8 × 10^8^ cfu ml^−1^, and treated with SDS–chloroform for *β*-galactosidase assay. Mean values of at least four independent trials were presented in Miller units with SD (error bars). ^**^P < 0.01.

In order to confirm this positive regulation of PrhN on *popA* expression, we constructed a *prhN* in-frame deletion mutant RK5601 (*popA-lacZYA, Δ prhN*) from RK5050 (OE1-1, *popA-lacZYA*) and evaluated *popA* expression. *popA* expression in RK5050 is seriously repressed in rich medium and well induced in sucrose medium (approximately 328 Miller Units). In sucrose medium, *popA* expression in the *prhN* mutant (RK5601) was significantly reduced to 15 Miller Units (Figure 1A, *p* < 0.01). The *prhN* mutant (RK5601) showed identically reduced *popA* expression as that of a transposon mutant. Strain RS1002 differs from OE1-1 for virulence in tobacco plants, and the *prhN* deletion in RS1002 also significantly diminished *popA* expression in sucrose medium (12 Miller Units) (Figure 1A, *p* < 0.01).

As *popA* expression is directly controlled by HrpB, we evaluated the effect of PrhN on *hrpB* expression. We constructed a *prhN* in-frame deletion mutant RK5604 (*hrpB-lacZYA, Δ prhN*) from RK5046 (OE1-1, *hrpB-lacZYA*) and evaluated *hrpB* expression in sucrose medium. *hrpB* expression in the *prhN* mutant (RK5604) was significantly reduced compared to that of RK5046 (19 vs. 173 Miller Units) (Figure 1B, *p* < 0.01). The complemented *prhN* gene (under the control of its native promoter) could completely restore *popA* and *hrpB* expression to that of wild-type (Figures 1A,B). As with the control, the empty vector (pUC18-mini-Tn7T-Gm) did not affect *popA* and *hrpB* expression (data not shown). All of these findings demonstrate that PrhN is important for the expression of *popA* and *hrpB* in *R. solanacearum in vitro*, and this control is not strain-specific.

### PrhN positively regulates *prhG* expression but is dispensable for *hrpG* expression

**Figure 2:**
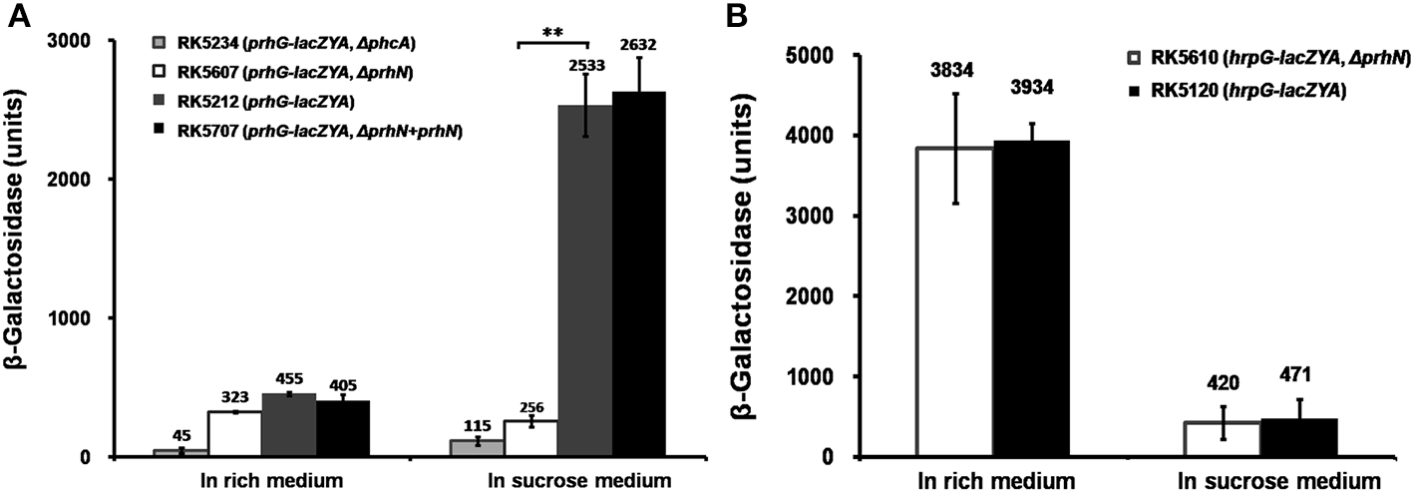
***prhG* and *hrpG* expression in *prhN* mutants. (A)** *prhG*, **(B)** *hrpG* expression in rich and sucrose media. **(A)**, RK5234 (*prhG-lacZYA*, Δ*phcA*), grayish bars; RK5607 (*prhG-lacZYA*, Δ*prhN*), white bars; RK5212 (*prhG-lacZYA*), gray bars and RK5707 (complementation, *prhG-lacZAY*, Δ*prhN*+*prhN*), dark bars. **(B)** RK5610 (*hrpG-lacZYA*, Δ*prhN*), white bars and RK5120 (*hrpG-lacZYA*), dark bars. Cells were grown to an OD600 of about 0.1 and enzyme activity was measured. Mean values of at least four independent trials were presented in Miller units with SD (error bars). ^**^P < 0.01.

As the transcription of *hrpB* is activated by both HrpG and PrhG, we evaluated the effect of PrhN on expression of *hrpG* and *prhG*. We constructed *prhN* in-frame deletion mutants RK5607 (*prhG-lacZYA, Δ prhN*) and RK5610 (*hrpG*-*lacZYA, Δ prhN*) from RK5120 (*hrpG*-*lacZYA*) and RK5212 (*prhG-lacZYA*), respectively. In sucrose medium, *prhG* expression in RK5212 (*prhG-lacZYA*) was approximately 2533 Miller units, and it was significantly reduced to 256 Miller units in RK5607 (*prhG-lacZYA, Δ prhN*) (Figure 2A, *p* < 0.01). Complemented PrhN could completely recover *prhG* expression to that of wild type (Figure 2A). Empty vector did not affect the *prhG* expression (data not shown). The *phcA* deletion substantially reduced *prhG* expression in both B and sucrose media (Zhang et al., 2013). In sucrose medium, *prhG* expression in the *phcA* mutant (RK5234) was even slightly lower than that in the *prhN* mutant (Figure 2A). In rich medium (B medium), RK5212 (*prhG-lacZYA*) and RK5607 (*prhG-lacZYA, Δ prhN*) exhibited almost equal *prhG* expression levels (Figure 2A). These results suggest that *prhG* expression is positively regulated by PrhN but only in sucrose medium. In both B and sucrose media, almost equal *hrpG* expression was observed in RK5120 (*hrpG*-*lacZYA*) and RK5610 (*hrpG*-*lacZYA, Δ prhN*) (Figure 2B), suggesting that PrhN is not involved in the regulation of *hrpG* expression in *R. solanacearum*.

### The expression of *prhN* increases with cell density and regulates *prhG* expression in parallel with PhcA

**Figure 3:**
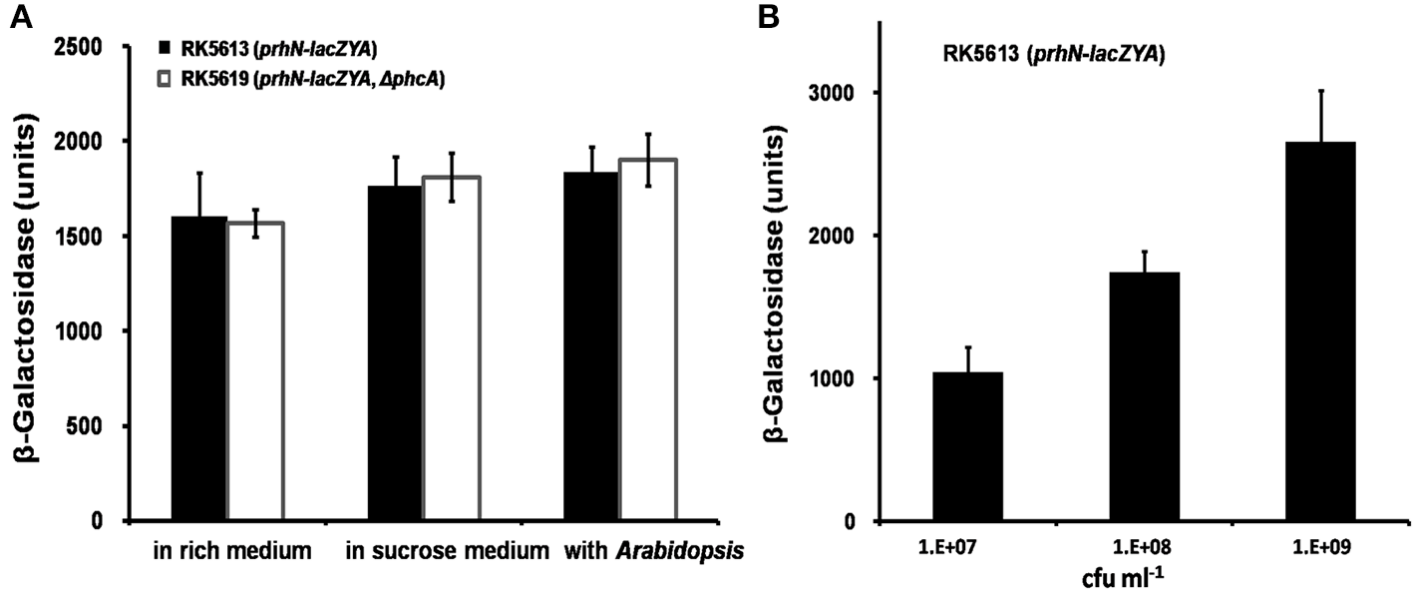
***prhN* expression under different conditions. (A)** *prhN* expression in *phcA* mutant in rich medium, sucrose medium and co-cultivation with *A. thaliana* seedlings. RK5613 (*prhN-lacZYA*), dark bars and RK5619 (*prhN-lacZYA*, Δ*phcA*), white bars. Cells were grown to an OD600 of about 0.1 and enzyme activity was measured. **(B)** *prhN* expression at different cell density in sucrose medium. Cells were grown to an OD600 of about 0.01, 0.1, and 1.0 (1 OD600 corresponds to 1.8 × 10^9^ cfu ml^−1^), and enzyme activity was measured. Mean values of at least four independent trials were presented in Miller units with SD (error bars).

As PhcA also positively regulates *prhG* expression (Zhang et al., 2013), we evaluated the effect of PhcA on *prhN* expression. We constructed a *prhN-lacZYA* reporter strain RK5613 (OE1-1 *prhN-lacZYA*) and a *phcA* deletion mutant RK5619 (*prhN-lacZYA, Δ phcA*) from RK5613. In rich medium, sucrose medium, and co-cultivation with *A. thaliana* seedlings, almost equal *prhN* expression levels were observed in RK5613 (*prhN-lacZYA*) and RK5619 (*prhN-lacZYA, Δ phcA*) (Figure 3A), suggesting that PhcA is not involved in the regulation of *prhN* expression in *R. solanacearum*. *phcA* mutants are well known to be deficient in extracellular polysaccharides (EPS) and show stick and matte cellular morphology. The *prhN* mutant remains mucoid and demonstrates abundant EPS production (data not shown), indicating that PrhN is dispensable for *phcA* expression in *R. solanacearum*. PhcA and PrhN should positively regulate *prhG* expression in a parallel way.

As *prhG* expression increases constantly with cell densities (Zhang et al., 2013), we evaluated *prhN* expression levels at different cell densities. In sucrose medium, *prhN* expression at approximate OD600s of 0.01, 0.1 and 1.0 reached 1045, 1743, and 2654 Miller units, respectively (Figure 3B). The expression profile of *prhN* with cell density was consistent with that of *prhG*.

### PrhN is important for full virulence of *R. solanacearum* in tomato plants but is not involved in HR elicitation

**Figure 4:**
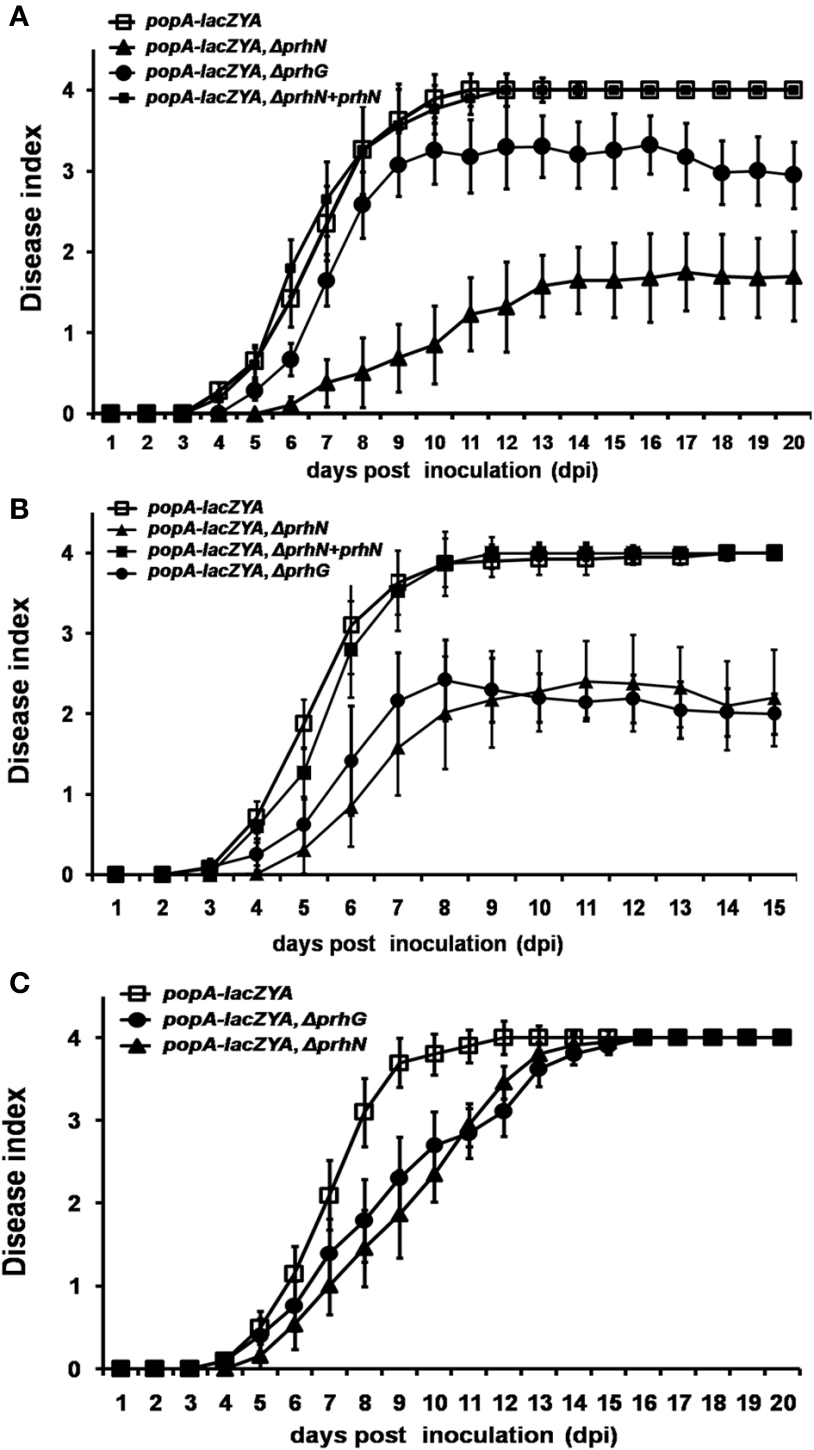
**Pathogenicity test of OE1-1 derived strains**. A bacterial suspension (10^8^ cfu ml^−1^) was poured onto the soil supporting growth of plants **(A)** tomato and **(C)** tobacco plants at a final concentration of 10^7^ cfu g^−1^ of soil. **(B)** 2μ l of bacterial suspension (10^8^ cfu ml^−1^) was dropped onto freshly cut surfaces of tomato petioles. RK5050 (*popA-lacZYA*), squares; RK5601 (*popA-lacZAY*, Δ*prhN*), closed triangles; RK5185 (*popA-lacZAY*, Δ*prhG*), closed circles; and RK5701 (complementation, *popA-lacZAY*, Δ*prhN*+*prhN*), closed squares. Plants were inspected daily for wilt symptoms, and scored on a disease index scale from 0 to 4 (0, no wilting; 1, 1–25% wilting; 2, 26–50% wilting; 3, 51–75% wilting; and 4, 76–100% wilted or dead). Each trial included at least 12 plants for inoculation with one strain and mean values of at least four independent trials were presented in Miller units with SD (error bars).

Since PrhN is important for *hrpB* and *prhG* expression in *R. solanacearum*, and *hrpB* and *prhG* are important for pathogenicity of *R. solanacearum* in host plants, we tested the effect of PrhN on virulence in host plants. When RK5050 (OE1-1, *popA-lacZYA*) was inoculated on host plants (tomato and tobacco plants), it showed virulence and bacterial growth *in planta* which were identical to those of the parental strain OE1-1 (Yoshimochi et al., 2009b; Zhang et al., 2011). Using soil-soak inoculation in tomato plants, which mimics natural infection from roots, RK5050 wilted tomato plants at 4 dpi and killed plants at 10 dpi (Figure 4A). Several tomato plants inoculated with the *prhN* mutant (RK5601) began to wilt at 6 dpi and died at 14 dpi. However, most plants remained alive up to 20 dpi (wilt symptoms reached approximately 1–2, Figure 4A). These results suggest that the *prhN* mutant exhibited less virulence than that of RK5050 in tomato plants. As reported previously (Zhang et al., 2013), the *prhG* mutant (RK5185) exhibited slightly less virulence than that of RK5050. The *prhN* mutant (RK5601) exhibited obviously less virulence that of the *prhG* mutant (RK5185) (Figure 4A). Complementation with PrhN (under the control of its native promoter) could completely restore the virulence of the *prhN* mutant (RK5601) to that of RK5050 (Figure 4A). As with the control, the empty vector (pUC18-mini-Tn7T-Gm) did not affect virulence (data not shown).

When tomato plants were challenged using petiole inoculation, which directly introduces cells into the xylem, reduced virulence was also observed with *prhN* mutant (RK5601), and it exhibited almost identical virulence to that of the *prhG* mutant (RK5185) (Figure 4B). Complementation with PrhN could completely recover the virulence of the *prhN* mutant to that of the wild-type strain (RK5050) (Figure 4B). The empty vector did not affect virulence (data not shown). We also evaluated the virulence of the *prhN* mutant (RK5601) in tobacco plants using soil-soaking inoculation. In contrast to the deleterious effect of the *prhN* deletion on bacterial virulence in tomato plants, *prhN* disruption only caused delayed symptom development in tobacco plants (Figure 4C).

**Figure 5:**
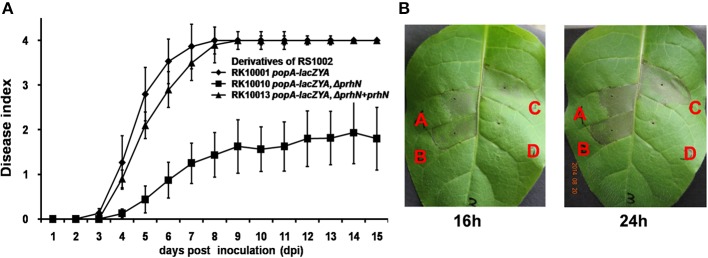
**Pathogenicity and HR test of RS1002 derived strains. (A)** Pathogenicity test in tomato plants. 2 μ l of bacterial suspension (10^8^ cfu ml^−1^) was dropped onto freshly cut surfaces of tomato petioles. RK10001(*popA-lacZYA*), closed rhombus; RK10010 (*popA-lacZYA*, Δ*prhN*), closed squares; and RK10013 (complementation, *popA-lacZAY*, Δ*prhN*+*prhN*), closed triangles. Each trial included at least 12 plants for inoculation with one strain and mean values of at least four independent trials were presented in Miller units with SD (error bars). **(B)** HR test in tobacco leaves. Approximately 50 μ l of bacterial suspension at 10^8^ cfu ml^−1^ was infiltrated into leaf mesophyll tissue with a blunt-end syringe. (A) RS1002; (B) RK10001 (*popA-lacZYA*); (C) RK10010 (*popA-lacZYA*, Δ*prhN*); and (D) distilled water. Pictures of tobacco leaves were taken at 16 and 24 h post infiltration (hpi). Each trial included four replications and three independent trials were performed. Results presented were from a representative experiment. Similar results were obtained in all other independent experiments.

We also tested the virulence of the *prhN* mutant (RK10010), (a derivative of RS1002, which causes disease in tomato plants and HR in tobacco leaves) in tomato plants. Using petiole inoculation, reduced virulence was also observed with the *prhN* mutant (RK10010) in tomato plants, and complementation with PrhN could completely restore its virulence to that of wild-type (Figure 5A). The empty vector did not affect virulence (data not shown). All of these results demonstrate that PrhN is important for full virulence of *R. solanacearum* in host plants, and it is not strain-specific.

Since RS1002 elicits a HR in tobacco leaves, we evaluated the effect of PrhN on HR elicitation of RS1002. We infiltrated *N. tabacum* BY leaves with RS1002, RK10001 (RS10002, *popA-lacZYA*) and RK10010 (RS1002, *popA-lacZYA, Δ phcA*), and evaluated the development of necrotic lesions. All three strains were able to cause necrotic lesions (Figure 5B). Lesions appeared at about 16 h post infiltration (hpi) and were maximal at about 24 hpi (Figure 5B). No obvious differences in the development of necrotic lesions were observed between these three strains, suggesting that PrhN is not involved in the HR elicitation by RS1002 in tobacco leaves.

### PrhN is involved in bacterial growth *in planta*

**Figure 6:**
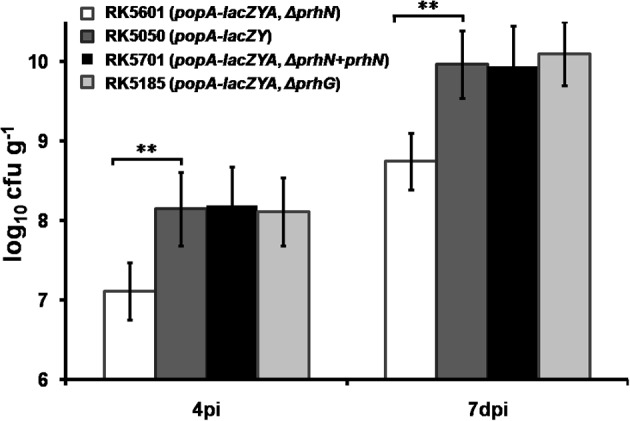
**Bacterial growth of *prhN* mutant in tomato stems**. Tomato plants were inoculated with bacteria using a soil-soak method at a final concentration of 10^7^ cfu g^−1^ of soil. Stem species (approximately 5 cm above the soil and 1 cm in length) were removed at 4 dpi (wilt symptoms reached 1–2) and 7 dpi (wilt symptoms reached 3–4). The cell number was quantified by dilution plating. RK5601 (*popA-lacZYA*, Δ*prhN*), white bars; RK5050 (wild type), grayish bars; RK5701 (complementation), dark bars; and RK5185 (*popA-lacZYA*, Δ*prhG*), gray bars. Each trial included at least six plants for inoculation with one strain and mean values of at least four independent trials were presented in RLU cell^−1^ with SD (error bars). ^**^P < 0.01.

The *prhN* mutant (RK5601) is less virulent in tomato plants and it exhibited similar bacterial morphology and growth profiles as did the wild-type strain in B and sucrose media (data not shown). Therefore, we evaluated the bacterial growth of the *prhN* mutant (RK5601) *in planta*. We inoculated tomato plants with RK5050 (*popA-lacZYA*), RK5185 (*popA-lacZYA, Δ prhG*), RK5601 (*popA-lacZYA, Δ prhN*), and RK5701 (*popA-lacZYA, Δ prhN+prhN*) by soil-soak and quantified bacterial cells in stem tissue. As reported previously, the *prhG* mutant (RK5185) and the wild-type strain (RK5050) can grow well in tomato stems and they proliferated in stem tissues to approximately 10^8^ cfu g^−1^ at 4 dpi (wilt symptoms reached ~1), and approximately 10^10^ cfu g^−1^ at 7 dpi (wilt symptoms reached ~3–4) (Figure 6). The *prhN* mutant (RK5601) proliferated in the stem to approximately 10^7^ cfu g^−1^ at 4 dpi and approximately 5 × 10^8^ cfu g^−1^ at 7 dpi. The *prhN* mutant (RK5601) proliferated *in planta* significantly less than did the wild-type bacteria at a difference of one to two orders of magnitude (*p* < 0.01). Complementation with PrhN could completely restore the bacterial growth of the *prhN* mutant (RK5601) to that of wild-type bacteria *in planta* (tomato plants). These results suggest that PrhN is a pathogenicity regulator and the growth deficiency of the *prhN* mutant *in planta* might be a cause of its reduced virulence.

### PrhN is important for *popA* expression in tomato plants but not in tobacco plants

**Figure 7:**
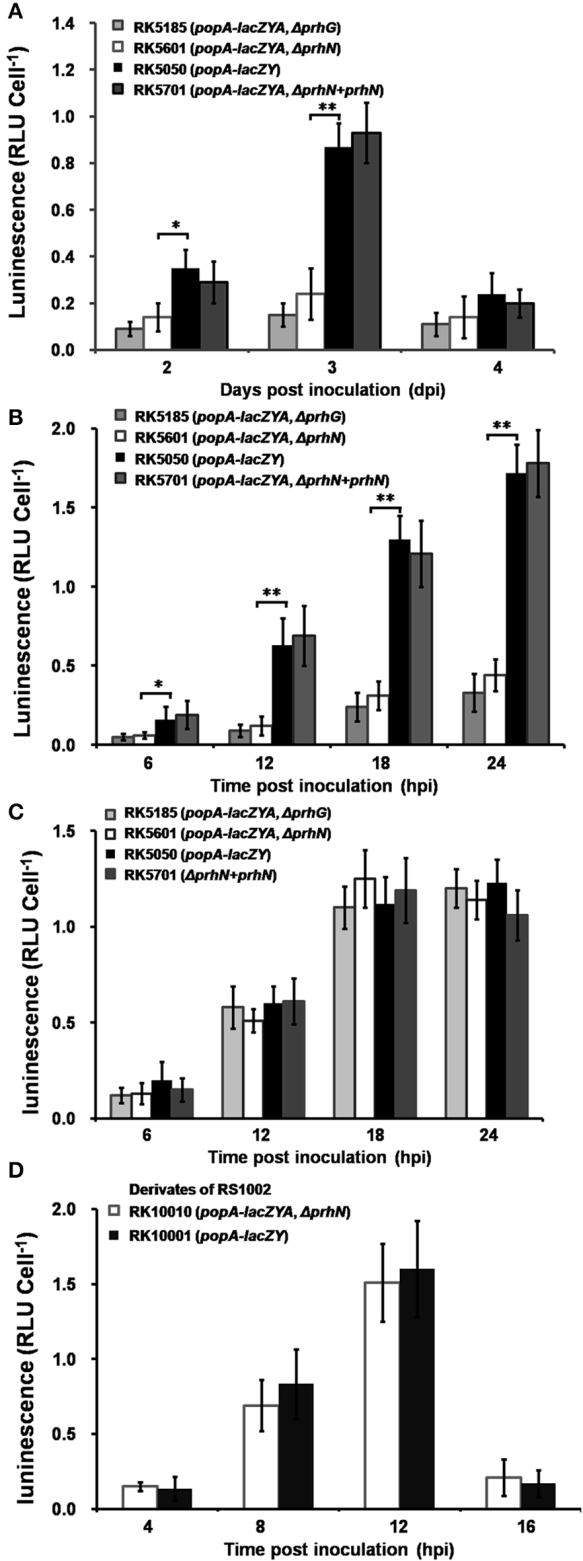
***popA* expression *in planta***. Strains RK5185, RK5601, RK5050, and RK5701 were inoculated into tomato stems using petiole inoculation method **(A)** and infiltrated into tomato leaves **(B)** and tobacco leaves **(C)**. Stem pieces were removed every day and leaf discs were removed every 6 hpi, and bacterial cells was measured using Galacto-Light Plus. Strains RK10001 and RK10010 were infiltrated into tobacco leaves **(D)**. Tobacco leaf discs were removed every 4 hpi, and bacterial cells was collected for *β*-galactosidase assay. Each assay included at least three samples from different plants and mean values of at least four independent trials were presented in RLU cell^−1^ with SD (error bars). ^*^P < 0.05; ^**^P < 0.01.

Since PrhG is involved in *popA* expression of OE1-1 in tomato plants (Zhang et al., 2013), we evaluated the effect of PrhN on *popA* expression *in planta* (tomato and tobacco). Strains RK5050 (wild-type), RK5185 (Δ*prhG*), RK5601 (Δ*prhN*), and RK5701 (Δ*prhN*+prhN) were used to inoculate tomato plants using petiole inoculation and bacterial cells were collected from stems periodically for *β*-galactosidase assay. Since *hrpB* and *hrpG* mutants completely lost virulence and bacterial growth *in planta*, we didn't use them for comparison with *prhG* and *prhN* mutants. At 1 dpi, cell numbers in the stem was low, and *popA* expression in the stem was undetectable. * Pop*A expression in RK5050 increased at 2 and 3 dpi, and then decreased to the basal level at 4 dpi. The *prhN* mutant showed significantly reduced *pop*A expression at 2 and 3 dpi compared to that of RK5050 (wild-type) in stem xylem vessels (Figure 7A, *p* < 0.05 at 2 dpi, and *p* < 0.01 at 3 dpi). As reported previously, the *prhG* mutant (RK5185) also showed significantly reduced *pop*A expression at 2 and 3 dpi, and is slightly lower than that of the *prhN* mutant (RK5601) (Figure 7A).

We also evaluated *popA* expression in leaf intercellular spaces. Strains RK5050 (wild type), RK5185 (Δ*prhG*), RK5601 (Δ*prhN*), and RK5701 (Δ*prhN*+prhN) were infiltrated into tomato and tobacco leaves and cells were recovered from infiltrated leaves for *β*-galactosidase assay. *PopA* expression in RK5050 was undetectable at time 0, and it increased at 12, 18, and 24 hpi. In tomato leaves, the *prhN* mutant (RK5601) showed significantly reduced *pop*A expression compared to that of RK5050 (Figure 7B, *p* < 0.05 at 6 hpi and *p* < 0.01 at 12, 18, 24 hpi). The *prhG* mutant (RK5185) also showed reduced *pop*A expression, but it is slightly lower than that of the *prhN* mutant (RK5601) in tomato leaves (Figure 7B). As reported previously (Zhang et al., 2013), the *prhG* mutant (RK5185) did not show a significant difference in *popA* expression in tobacco leaves from that in wild-type (Figure 7C). In contrast to tomato leaves, no significant difference was observed between the *prhN* mutant (RK5601) and wild-type bacteria in tobacco leaves (Figure 7C).

Since RK10001 and RK10010 (Δ*prhN*) showed almost equal HR elicitation in tobacco leaves, we evaluated *popA* expression in a *prhN* mutant (RK10010) in tobacco leaves. RK10001 (wild-type) or the *prhN* mutant (RK10010) were infiltrated into tobacco leaves and *pop*A expression was evaluated. Necrotic lesions appeared at 16 hpi in tobacco leaves, indicating bacterial cells began to die at this time, and therefore we collected bacterial cells from infiltrated tobacco leaves every 4 h from 4 hpi. *Pop*A expression in RK10001 increased at 4, 8, and 12 hpi, and then decreased quickly at 16 hpi. The *prhN* mutant (RK10010) showed almost identical *popA* expression levels as that in wild-type bacteria (Figure 7D), reflecting that the *prhN* mutant is equivalent in HR elicitation in tobacco leaves to that in wild-type bacteria. These results suggest that PrhN is a regulator of *popA* expression in tomato plants, and that the reduced *popA* expression of *prhN* mutants *in planta* might be another cause of its reduced virulence in tomato plants, but this control is host plant-specific.

## Discussion

The MarR regulator was originally identified as repressor of multiple antibiotic resistance in *E coli*, and variants are widely distributed in bacteria and involved in the regulation of various cellular processes, including pathogenesis by both plant and human pathogens (Alekshun et al., 2001; Ellison and Miller, 2006). In the present study, we have genetically demonstrated that *prhN* (*rsc1721* in GMI1000 that encodes a putative MarR-type regulator) is involved in the pathogenesis of *R. solanacearum* in tomato plants. MarR members control expression of some virulence-related genes or virulence associated traits; however, their regulatory targets vary in different pathogens (Ellison et al., 2004; Wilkinson and Grove, 2004).

In *R. solanacearum*, the *hrp* regulon and its encoding T3SS are essential for pathogenicity in host plants and for HR elicitation in resistant or non-host plants (Van Gijsegem et al., 1995; Coll and Valls, 2013). The expression of the *hrp* regulon and type III effectors are directly controlled by HrpB. HrpB controls multiple virulence pathways, including chemotaxis, biosynthesis or catabolism of various low-molecular-weight chemical compounds, and siderophore production and uptake (Jacobs et al., 2012; Coll and Valls, 2013). The expression of *hrpB* is activated by both HrpG and PrhG in a cell density-dependent manner both *in vitro* and *in planta* (Plener et al., 2010; Zhang et al., 2013). HrpB and HrpG are well known to be master regulators since *hrpB* and *hrpG* mutants completely lose virulence and bacterial growth in host plants (Vasse et al., 2000; Yoshimochi et al., 2009b). Despite the high similarity (72% global identity), PrhG displays a quite distinct role from HrpG. The *prhG* mutant exhibits equivalent bacterial growth *in planta* as do wild-type bacteria and is just slightly less virulent compared to that of wild-type bacteria in tomato plants (Plener et al., 2010; Zhang et al., 2013). Another remarkable difference between PrhG and HrpG is that their expression is positively and negatively regulated by PhcA, respectively. In this study, we genetically demonstrated that PrhN positively regulates *prhG* expression (only in *hrp*-inducing conditions), but it is dispensable for *hrpG* expression. This, in turn, results in the positive regulation of the expression of *hrpB* and *popA* (representative of a T3SS and the *hrp* regulon). *PrhG* expression increases constantly with cell density and is substantially reduced in *phcA* deletion mutants in both rich medium and *hrp*-inducing medium (Zhang et al., 2013). Although, PrhN positively regulates *prhG* expression only in *hrp*-inducing conditions, the expression manner of *prhG* at different cell densities is consistent with that of *prhG*. It was previously reported that PhcA positively regulates *prhG* expression (Zhang et al., 2013), while PhcA does not control *prhN* expression. The *phcA* deletion caused substantially reduced *prhG* expression (45 vs. 455 Miller units in rich medium and 115 vs. 2533 Miller units in *hrp*-inducing condition). The *prhN* deletion reduced *prhG* expression to 256 Miller units only in *hrp*-inducing conditions (Figure 2A). *PrhG* expression in *prhN* mutants was slightly higher than that in *phcA* mutants in *hrp*-inducing conditions (Figure 2A). Moreover, *prhN* mutants retain the same cellular morphology as wild-type bacteria (typical abundant mucoid EPS), and are totally different from that of *phcA* mutants, which usually results in a non-mucoid colony morphology because of deficient EPS production. PhcA and PrhN positively regulate *prhG* expression in a parallel way. HrpG is well known to be involved in the pathway triggered by plant signals or some mimic plant signals. PrhG is mainly involved in pathways triggered by metabolites and cell density (Plener et al., 2010). *prhN* expression maintained an almost equivalent level in rich medium, *hrp*-inducing medium, and co-cultivation with *A. thaliana* seedlings (Figure 3A), suggesting that PrhN may be not involved in the response to different environmental conditions.

It was previously reported that the expression of *popA* and *hrpB* was also reduced in *prhG* mutants *in planta* (Zhang et al., 2013). Here we report that PrhN is also important for the expression of *popA* and *hrpB in vitro* and *in planta* (only in tomato plants). Moreover, PrhG was dispensable for bacterial growth *in planta*, while the *prhN* mutant proliferated in tomato stems significantly less efficiently (one or two orders of magnitude) than wild-type bacteria (Figure 6). These results suggest that PrhN might control not only *prhG* expression (and in turn the *hrp* regulon) but also some unknown pathogenicity determinants. As reported previously, the *prhG* mutant was slightly less virulent than were wild-type bacteria when challenged tomato plants using soil-soaking inoculation, and it is obviously less virulent than wild-type bacteria on challenged tomato plants using petiole inoculation (Figures 4A,B). The *prhN* mutant always exhibited less virulence than wild-type bacteria when tomato plants were challenged using both methods. Although, the *prhN* mutant exhibited almost equivalent virulence as did the *prhG* mutant when tomato plants were challenged using petiole inoculation (Figure 4B), the *prhN* mutant exhibited much less virulence than did the *prhG* mutant when tomato plants were challenged using soil-soaking inoculation (Figure 4A). *PrhG* expression is positively controlled by PhcA, which becomes active in a cell density-dependent manner (Clough et al., 1997). PrhG mainly functions at high cell density (Zhang et al., 2013). *PrhN* expression increased constantly with cell density, and it was not regulated by PhcA, suggesting that PrhN might function during the whole infection process. Taken together, these data may explain why the *prhN* mutant exhibited less virulence than do wild-type bacteria in tomato plants, while the *prhG* mutant just exhibited less virulence on tomato plants using petiole inoculation.

In tobacco plants, PrhN contributes little to *popA* expression and disease development in strain OE1-1, suggesting that PrhN might play different roles in disease development in different plants. PrhN positively regulates *prhG* expression and PrhG was also reported to play different roles in disease development in different plants (Zhang et al., 2013). The contribution of PrhG and PrhN to virulence is consistent in tobacco plants. In strain OE1-1, it was also reported that *prhK*, *prhL*, and *prhM* mutant strains completely lost pathogenicity toward tomato plants, while they were slightly less virulent toward tobacco plants (Zhang et al., 2011). It seems common that some virulence regulators in OE1-1 play different roles in disease development on different plants.

Another *R. solanacearum* strain RS1002 causes disease in tomato and HR in tobacco leaves. Here, we provide evidence that PrhN does play an important role in disease development of RS1002 in tomato plants. Similar to strain OE1-1, *prhN* mutants in RS1002 exhibited significantly reduced *popA* expression under *hrp*-inducing conditions and obviously reduced virulence in tomato plants. In tobacco leaves, RS1002 and the *prhN* mutant showed almost equivalent *popA* expression level. This result might be able to explain why RS1002 and its *prhN* mutant showed almost identical HR development in tobacco leaves.

MarR family members display various biological functions in many bacteria and archaea. Some MarR family members are involved in the detoxification of aromatic compounds and the regulation of the expression of ferulic catabolic genes (Fiorentino et al., 2007; Calisti et al., 2008). In *D. dadantii* 3937, the MarR member MfbR is involved in the modulation of virulence gene expression in response to an acidic pH (Reverchon et al., 2009). In *X. campestris* pv. campestris, the MarR regulator HpaR is important for the production of an extracellular protease in *hrp*-inducing medium. HpaR is dispensable for the production of extracellular endoglucanase and amylase (Zou et al., 2012). Regulatory targets of MarR members usually vary in different pathogens (Perera and Grove, 2010). The regulatory mechanism of the MarR-type regulator PrhN remains unclear. We plan to elucidate further various biological functions of PrhN in *R. solanacearum*. We have genetically demonstrated that the newly characterized MarR-type regulator PrhN is important for *hrp* expression and full virulence of *R. solanacearum* in tomato plants. PrhN positively regulates the expression of *prhG*, *hrpB* and *popA*, but it is dispensable for the expression of *hrpG* and *phcA*. This finding will improve our understanding of complex regulatory pathway of the *hrp* regulon in *R. solanacearum* and the various biological functions of MarR regulators.

### Conflict of interest statement

The authors declare that the research was conducted in the absence of any commercial or financial relationships that could be construed as a potential conflict of interest.
